# Peculiarities of the regional dynamics of the prevalence and incidence of cystitis in children

**DOI:** 10.25122/jml-2020-0121

**Published:** 2021

**Authors:** Volodymyr Volodymyrovych Bezruk, Tetyana Oleksandrivna Bezruk, Oleksii Serhiiovych Godovanets, Michael Ivanovych Sheremet, Svyatoslava Vasylivna Yurniuk, Mariia Ivanivna Velia, Olena Viktorivna Makarova, Oksana Ivanivna Yurkiv, Oleh Olehovych Maksymiv

**Affiliations:** 1. Department of Pediatrics, Neonatology and Perinatology Medicine, Bukovinian State Medical University, Chernivtsi, Ukraine; 2. Department of Internal Medicine and Infectious Diseases, Bukovinian State Medical University, Chernivtsi, Ukraine; 3. Surgery Department No.1, Bukovinian State Medical University, Chernivtsi, Ukraine; 4. Department of Pharmacy, Bukovinian State Medical University, Chernivtsi, Ukraine; 5. Department of Care for Patients and Higher Nursing Education, Bukovinian State Medical University, Chernivtsi, Ukraine; 6. Department of Orthopedic Dentistry, Bukovinian State Medical University, Chernivtsi, Ukraine.

**Keywords:** chronic cystitis, children, medical statistics

## Abstract

In a child, cystitis (non-specific microbial inflammation of the mucous membrane of the bladder) is considered to be a dangerous disease; the prolongation of the process is usually associated with a delayed diagnosis. The aim of this work was to analyze the health status of the child population of the Chernivtsi region, especially the dynamics of the prevalence and incidence of cystitis. The official statistical data have been studied (reports on the state of medical care for children in the Chernivtsi region and data from the Center of Medical Statistics of the Ministry of Healthcare from 2006 to 2017); information-analytical and statistical methods have been used for the purpose of this study. Attention should be drawn to the significantly high prevalence of cystitis among children aged 15–17 years, especially in the Chernivtsi region as during period I (8.7±0.6 vs. 4.3±0.3 in Ukraine) and II (11.7±1.0 and 5.7±0.4, respectively, per 1000 people). Moreover, over the years, the growth of indicators acquires intensity, while this process is more than twice as pronounced in Chernivtsi. Thus, the growth rate was 65.0% in 2006–2011 and 90.3% in 2012–2017 vs. 27.2% and 32.8% in Ukraine, respectively. The identified data indicate the need to provide specialized care to children with infectious and inflammatory diseases of the urinary system of the Chernivtsi region and the need to improve regional clinical routes of patients with infectious and inflammatory diseases of the urinary system.

## Introduction

Cystitis, or inflammation of the bladder, affects the bladder function directly. Both infectious and noninfectious etiologies are possible. Gram-negative bacteria such as Proteus, Klebsiella, Citrobacter, Enterobacter, and Pseudomonas species and Gram-positive pathogens such as Enterococcus fecalis, Staphylococcus saprophyticus, and group B streptococci are able to cause an infection. However, Escherichia coli represents the most common cause of infectious cystitis [1–3]. Noninfectious cystitis can be due to a variety of causes, such as medication, radiation, foreign bodies, chemicals, autoimmune response, and may even be idiopathic – interstitial cystitis (IC). It may also occur in association with other diseases such as gynecological cancer, pelvic inflammatory disease (PID), and Crohn’s disease. Irrespective of the cause, cystitis can be acute or chronic depending upon the duration of the insult [4–6].

The first and early response to any harmful stimulus or injury occurs in the form of acute inflammation. Acute inflammation is defined by vasodilation and increase of vascular permeability, leukocyte migration to the site of damage, and activation of the biochemical cascade of inflammation, resulting in the release of mediators such as cytokines, histamines, kinins, complement factors, nitric oxide, clotting factors, and proteases [7]. In acute cystitis, these mediators cause erythematous swelling and ulceration of the bladder mucosa, which bleeds easily [8]. The surface layer is shred, forming small, clear cysts (sacs with liquid, gas, or semisolid contents), which are frequently seen on urine analysis [9–12].

Besides, these mediators produce irritation of the bladder mucosa, which is responsible for urgency, increased frequency, and dysuria [13]. Also, the systemic release of inflammatory mediators causes low-grade fever. However, these mediators have a short half-life and are quickly degraded, enabling rapid inflammation resolution as soon as the noxious stimulus is removed [14–17].

However, if the stimulus is not removed, chronic inflammation ensues, as seen in IC [18–20], which is characterized by infiltration of mononuclear cells such as macrophages, lymphocytes, eosinophils, mast cells, and plasma cells, leading to irreversible tissue destruction, dysfunctional pathology such as fibrosis and poor compliance, detrusor overactivity, and hyperalgesia responsible for the chronic waxing and waning symptoms of pain and lower urinary tract symptoms.

## Material and Methods

The aim of this work was to analyze the health status of the child population of the Chernivtsi region, especially the dynamics of the prevalence and incidence of cystitis. The official statistical data have been studied (reports on the state of medical care for children in the Chernivtsi region and data from the Center of Medical Statistics of the Ministry of Healthcare from 2006 to 2017). This work is a fragment of the scientific and research paper: “Scientific support, monitoring and evaluation of models of health care development in Ukraine at the regional level” (due date 2015–2017), state registration no. 0115U002852. To substantiate the provisions and identification of priority approaches to optimize and improve the quality of medical care at the regional level, the peculiarities of the dynamics of the prevalence and incidence of cystitis among the child population of the Chernivtsi region have been analyzed.

Statistical data were calculated and compared using the MedCalc software, developed by “MedCalc Software” (Ostend, Belgium). The immunohistochemical data are reported as mean±SEM. The data obtained were statistically processed using the Mann-Whitney U test. Bivariate correlation between variables was determined by Pearson’s correlation coefficients. A p-value <0.05 was considered significant. Results are represented as mean±SD.

## Results

The results of the analysis obtained in the course of the study showed the existing regional peculiarities of the dynamics of the prevalence and incidence of cystitis among the children of the Chernivtsi region and their difference from the all-Ukrainian ones [7, 22, 23]. One of these features results from Table 1, i.e., in the Chernivtsi region, the percentage of children aged 0–14 years with cystitis is two times greater compared to the number of children from the whole country. Among adolescents, the difference between their values reached validity (1.6±0.5% vs. 0.7±0.09%, respectively).

**Table 1.** The specific weight of patients with cystitis – the total number of children from Chernivtsi and Ukraine as a whole, 2017.

**Territory**	**0–14 years old**		15–17 years old		Total	
	**%**	**m**	**%**	**m**	**%**	**m**
**Ukraine**	0.24	0.038	0.7	0.09	0.3	0.036
**Chernivtsi region**	0.45	0.25	1.6 *	0.52	0.6	0.22

* the difference in the age group is significant; p<0.05.

The analysis of absolute values, the dynamics of which according to the study periods is given in Table 2, demonstrates, in contrast to the general Ukrainian tendency, a significant increase in adolescents registered in the Chernivtsi region and which is growing with each subsequent period (by 40.7% and 51.0% in the period I and II, respectively). No significant changes in the dynamic nature of the number of registered children with cystitis at the age of 0–14 years were observed. Simultaneously, these age-related processes in the Chernivtsi region affected the overall state of accumulation of the specified category of children.

**Table 2.** Dynamics of registered patients with cystitis among the child population of Ukraine and the Chernivtsi region.

Years	Ukraine			Chernivtsi region		
	0–14 years old	15–17 years old	Total	0–14 years old	15–17 years old	Total
**2006**	17879	7868	25747	789	253	1042
**2011**	16454	7399	23853	746	356	1102
**Dynamics, % for period I**	-8.0	-6.0	-7.4	-5.4	+40.7	+5.7
**2012**	16133	7197	23330	711	304	1015
**2017**	15850	7169	23019	713	459	1172
**Dynamics, % for period II**	-1.7	-0.4	-1.3	+0.3	+51.0	+15.5

The study of the dynamic series of cystitis prevalence among children of different age groups, undoubtedly, gives objective information about the situation in the Chernivtsi region in a comparative aspect with the dynamics of the process which takes place in Ukraine as a whole (Table 3).

**Table 3.** Prevalence dynamic of cystitis among children in Ukraine and the Chernivtsi region (per 1000 people of the corresponding population).

Years Periods	Ukraine		Chernivtsi region	
	0–14 years old	15–17 years old	0–14 years old	15–17 years old
	1	2	3	4
**2006**	2.64	3.86	4.98	6.03
**2011**	2.53	4.91	4.98	9.95
**M±m for Period I**	2.56±0.05	4.3±0.3 *	5.0±0.3 *	8.7±0.6 *
**Dynamics, % for period I**	-4.2	+27.2	-	+65.0
**2012**	2.47	5.00	4.75	8.64
**2017**	2.43	6.64	4.56	16.44
**M±m for Period II**	2.43±0.03	5.7±0.04 Δ *	4.42±0.2 *	11.7±1.0 Δ *
**Dynamics, % for period II**	-1.6	+32.8	-4.6	+90.3

* the difference is significant between the P _2-4, 1-2, 3-4, 1-3_ indicators; p<0.05;

Δ the difference is significant between the indicators according to the periods; p<0.05.

First of all, according to the presented data, there is a significantly high prevalence of cystitis among children aged 15–17 years, especially in the Chernivtsi region during period I (8.7±0.6 vs. 4.3±0.3 in the country), and II (11.7±1.0 and 5.7±0.4, respectively, per 1000 people). Moreover, over the years, the growth of indicators acquires intensity, while this process is more than twice as pronounced in the Chernivtsi region. Thus, the growth rate was 65.0% in 2006–2011 and 90.3% in 2012–2017 in the Chernivtsi region vs. 27.2% and 32.8% in the rest of the country, respectively. Attention is drawn to the indicators among children aged 0–14 years. In the Chernivtsi region, they are also significantly higher than in the other regions of Ukraine; the dynamic nature of their changes is similar to an intensive decrease. There is an unfavorable picture in the Chernivtsi region, i.e., the accumulation of adolescents with cystitis in need of specialized medical care, which is confirmed below in the analysis of Table 4. It presents the dynamics of the incidence of various age groups of children with cystitis.

**Table 4.** Dynamics of the incidence of cystitis in children in Ukraine and the Chernivtsi region (per 1000 people of the corresponding population).

Years Periods	Ukraine		Chernivtsi region	
	0–14 years old	15–17 years old	0–14 years old	15–17 years old
	1	2	3	4
**2006**	2.48	3.67	4.78	5.82
**2011**	2.53	4.64	4.98	9.58
**M±m for Period I**	2.44±0.06	4.1±0.31 *	4.7±0.19	8.58±2.8 *
**Dynamics, % for period I**	+2.0	+26.4	+4.2	+64.6
**2012**	2.32	4.70	4.75	8.27
**2017**	2.29	6.35	4.18	15.83
**M±m for Period II**	2.28±0.04	5.43±0.56 Δ *	4.31±0.23	11.1±2.59 Δ *
**Dynamics, % for period II**	-1.3	+35.1	-12.0	+34.2

* the difference is significant between the P _2-4, 1-2, 3-4, 1-3_ indicators; p<0.05;

Δ the difference is significant between the indicators according to the periods; p<0.05.

When comparing the data of Tables 3 and 4, first of all, in the context of this provision, it should be indicated that the rate of increase in prevalence levels is higher than the incidence rate. Therefore, the increase in the number, especially teenagers, is not due to first-time cases. This is the difference between the situation in the Chernivtsi region and the country as a whole, where, on the contrary, the growth rate of incidence surpasses the prevalence. Thus, during 2011–2017, the increase in prevalence in the region was 90.3%, the incidence was 34.2%, while in the country, the rates were 35.1% and 32.8%, respectively. In other ways (in terms of values, age characteristics), the compared phenomena are close to each other. In particular, as in the case of prevalence, the incidence rates are higher among adolescents everywhere; they are growing significantly over the study periods. In addition, their values are significantly higher among all age groups in the Chernivtsi region than the average in Ukraine. In total, more than 1000 children are detected with the first established diagnosis in the region every year.

The second considerate fact that confirms the insufficient level of medical care in this category of patients is the specific weight of chronic cystitis cases (Table 5).

**Table 5.** Dynamics of the specific weight of chronic cystitis cases among different age groups of the child population of Ukraine and the Chernivtsi region by years of study.

Years	Ukraine			Chernivtsi region		
	0–14 years old	15–17 years old	Total	0–14 years old	15–17 years old	Total
**2006**	8.5	8.0	8.3	7.6	6.0	7.2
**2011**	9.3	8.0	8.9	7.4	4.2	6.4
**Dynamics, % for period I**	+9.4	-	+7.2	-2.6	-30.0	-11.1
**2012**	8.4	9.8	8.8	7.5	5.3	6.8
**2017**	6.9	6.0	6.6	12.0	7.8	10.3
**Dynamics, % for period II**	-17.9	-38.8	-25.0	+60.0	+47.2	+51.5

In contrast to the previous six years of study, during 2012–2017, the growth rate of patients with chronic cystitis among the child population of the Chernivtsi region was 51.5%, which is radically different from the data characteristic of the country, where they were less than a quarter. The difference is that against the background of a negative process (-17.9%) in the latter among children aged 0–14 years in the Chernivtsi region, the growth rate was 60.0%. Among teenagers, the values were (– 38.8%) and (+47.2%), respectively.

The data obtained for absolute values are similar to the nature of changes with intensive ones. Thus, for period II, the prevalence of chronic cystitis among children aged 0–14 years in the Chernivtsi region has significantly increased, namely up to 0.39±0.02 from 0.28±0.01 in period I against their decrease in the country – 0.18±03 and 0.22±0.06, respectively (per 1000 people). From the presented data, it is clear that if the indicators did not differ from each other during the first period, they were significantly higher in the Chernivtsi region during the second period. This feature can be traced among teenagers. The mean value of the prevalence level of chronic cystitis was 0.3±0.08 and 0.4±0.05 in the country and the Chernivtsi region in 2006–2011 (p>0.05). In 2012–2017, the values were 0.41±0.04 and 0.85±0.3, respectively(p<0.05) per 1000 people of the corresponding population).

The most dangerous and, at the same time, the most cases of common chronic cystitis are found among females [3, 5, 6, 13, 16, 20, 22]. Table 6 presents the specific weight of girls with chronic cystitis among the total number of such patients. The data analysis confirms the known situation, indicates the growth of the indicator, more intense in the first six years in Ukraine and the Chernivtsi region in particular. However, in each period, it was more pronounced in the Chernivtsi region.

**Table 6.** The percentage of girls presenting with chronic cystitis in dynamics in Ukraine and the Chernivtsi region.

Years	Ukraine			Chernivtsi region		
	0–14 years old	15–17 years old	Among all	0–14 years old	15–17 years old	Among all
**2006**	68.6	63.4	67.05	53.3	60.0	54.7
**2011**	80.5	82.8	81.2	74.5	81.3	77.1
**Dynamics, % for period I**	+18.8	+30.6	+21.0	+21.2	+35.5	+38.8
**2012**	84.0	82.1	83.3	86.8	87.5	87.0
**2017**	85.3	84.0	85.0	89.4	91.7	90.1
**Dynamics, % for period II**	+1.5	+2.3	+2.0	+3.0	+4.8	+3.6

Congenital abnormalities of the urinary system, kidney defects are followed, combined, and complicated by urinary tract infections. Therefore, for a complete picture of the situation in the Chernivtsi region, we analyzed the indicators of incidence and prevalence of pathology among the child population of the region. For a general idea, we shall imagine what percentage corresponds to children of different age groups with these pathologies considering their number in the region and the country (Table 7).

**Table 7.** The specific weight of children with congenital abnormalities of the urinary system and kidney defects considering the total number of children from Ukraine and the Chernivtsi region, 2017.

Pathology	Ukraine			Chernivtsi region		
	0–14 years old	15–17 years old	Total	0–14 years old	15–17 years old	Total
**Congenital abnormalities of the urinary system**	0.26	0.31	0.24	0.28	0.53	0.32
**Congenital kidney defects**	0.21	0.30	0.22	0.27	0.49	0.30
**Total**	0.47	0.61	0.46	0.55	1.02	0.62

Data presented in Table 7 show that the problem of providing specialized care for children with birth defects is topical in the Chernivtsi region.

## Discussion

Clinically, pediatric urinary tract infections (UTI) presentations are challenging because symptoms are vague and variable [1–4]. Young infants may present with sepsis or fever and lack specific symptoms, whereas older children present with classical features such as dysuria, frequency, and loin pain [2–8]. Early diagnosis with appropriate urine specimen collection techniques, investigations, and treatment is necessary to prevent renal damage and recurrence [3–6]. Effective, evidence-based investigations and treatment options are available, and physicians should feel confident in identifying and managing pediatric urinary tract infections (UTIs) [9–11].

UTIs are common in childhood. An estimated 2% of boys and 8% of girls will experience a UTI by seven years of age, and 7% of febrile infants have UTI [7, 21]. Pediatric UTIs, especially in young children, have various and non-specific presentations that can be undetected or misdiagnosed. Delaying diagnosis and management of UTIs may result in renal damage and renal function loss [14,22]. The aim of this article is to provide clinicians with an overview of the assessment and management of children with UTIs.

After all, a larger percentage of patients is concentrated here relative to the population and compared to the average value of the indicator in Ukraine [16, 22, 23], and this concerns adolescents and indicates that they were not treated during an earlier period. Moreover, this is evidenced by the ratio of incidence rates, which are 1.5 times higher among children aged 0–14 years (for example, 0.28 vs. 0.18 among adolescents in 2017 per 1000 people of the corresponding population). At the same time, the opposite picture appears when analyzing the prevalence; in this case, its level is almost twice as high among adolescents (5.30 vs. 2.81 among children aged 0–14 years, per 1000 people of the corresponding population). In addition, their values are growing over the years – by 45.0% for the first period and 26.2% for the second.

## Conclusions

In the Chernivtsi region, the percentage of children aged 0-14 years with cystitis among its total number is twice as high as in Ukraine. Also, it is even higher among adolescents, and the numbers are continuosly increasing, while a decrease similar to the national tendency is seen in children aged 0-14 years old. The identified data indicate the lack of clear mechanisms of interaction between primary, secondary, tertiary, outpatient and inpatient care in providing specialized medical care to children with infectious and inflammatory diseases of the urinary system of the Chernivtsi region and the need to improve regional clinical routes of patients with infectious and inflammatory diseases of the urinary system, especially in patients with cystitis.

## Acknowledgments

### Conflict of interest

The authors declare that there is no conflict of interest.
